# Pentamethylquercetin induces adipose browning and exerts beneficial effects in 3T3-L1 adipocytes and high-fat diet-fed mice

**DOI:** 10.1038/s41598-017-01206-4

**Published:** 2017-04-25

**Authors:** Yi Han, Jian-Zhao Wu, Ji-zhong Shen, Lei Chen, Ting He, Man-wen Jin, Hui Liu

**Affiliations:** 1 Department of Pharmacology, School of Basic Medicine, Tongji Medical College, Huazhong University of Science and Technology, Wuhan, China; 2 Department of Pharmacy, Shandong Provincial Qianfoshan Hospital, Shandong University, Jinan, China; 3 The Key Laboratory for Drug Target Researches and Pharmacodynamic Evaluation of Hubei Province, Wuhan, China; 4 Biomedicine Research Center, Wuhan Institute of Biotechnology, Wuhan, China

## Abstract

Browning white adipocytes may be a new target in anti-obesity therapy. Pentamethylquercetin (PMQ) has been shown to have anti-obesity effects in monosodium glutamate-induced obese mice. Here, we aimed to study the anti-obesity effects of PMQ *in vitro* and *in vivo* and to determine if adipose browning is involved in the mechanism underlying the anti-obesity effects of PMQ. We evaluated the effects of PMQ on cell proliferation, cell differentiation, glucose consumption, cellular lipid metabolism, and related brown gene expression in 3T3-L1 adipocytes. We also investigated the effects of PMQ in a mouse model of high-fat diet (HFD)-induced obesity. Our results demonstrated that PMQ increased the consumption of glucose, inhibited the accumulation of cellular triglycerides (TGs), and induced the expression of brown adipocyte-specific genes, such as uncoupling protein 1 (UCP-1), during the early stage of differentiation in 3T3-L1 adipocytes. In HFD mice, PMQ treatment reduced waist circumference, LEE index, white adipose tissue (WAT) weight and white adipocyte size and increased brown adipose tissue (BAT) weight. Moreover, PMQ treatment induced mitochondrial biogenesis and upregulated UCP-1 expression in WAT. These findings suggest that PMQ may induce browning of adipose tissue, a phenomenon that is at least partly related to its anti-obesity effects.

## Introduction

Obesity is becoming a worldwide health threat. Without effective intervention, 38% of the world’s adult population will be overweight, and another 20% of the population will be obese by the year 2030^1^. Obesity is closely linked to metabolic disorders, such as type 2 diabetes and metabolic syndrome, as well as other diseases, such as atherosclerosis and hyperlipidemia^2^. Obesity results from an imbalance between energy intake and expenditure. The best treatment for obesity is lifestyle modification, which is difficult to sustain. Medicines also are options for people in whom lifestyle changes are insufficient or ineffective. The United Stated Food and Drug Administration has recently approved several new anti-obesity drugs; however, the efficacies and adverse effects of these drugs remain to be evaluated further^3^. Therefore, new, safer and more effective drugs and therapeutic targets are still needed in weight loss therapy.

All mammals possess the following two distinct types of adipose tissue: white adipose tissue (WAT) and brown adipose tissue (BAT). WAT stores energy in the form of triglycerides (TGs) and releases hormones and cytokines that modulate whole-body metabolism and insulin resistance^4^. In contrast, BAT has abundant mitochondria, which possess the characteristic protein uncoupling protein 1 (UCP-1). UCP-1 is capable of directly dissipating energy as heat by uncoupling the electron transport chain from ATP synthesis^5, 6^. Obesity development depends not only on the balance between food intake and caloric utilization but also on the balance between WAT, the main energy reservoir, and BAT, which functions specifically in energy expenditure by facilitating nonshivering thermogenesis through UCP-1^7^. BAT thus represents a natural target for energy expenditure modulation and may be useful as a target for pharmacologic interventions^8^. Many studies have shown that inducing UCP-1 expression and brown adipogenesis in WAT can reverse genetic obesity in animal models^9, 10^. Thus, pharmacologic interventions facilitating the recruitment of brown fat cells or the browning of white fat cells would represent a new direction in obesity therapy^11^.

3,3′,4′,5,7-Pentamethylquercetin (PMQ) is a natural polymethoxylated flavonoid present in seabuckthorn (Hippophae rhamnoides)^12^. We have synthesized PMQ and demonstrated that the chemical has cardioprotective effects^13, 14^. Our previous study also showed that PMQ can upregulate peroxisome proliferator-activated receptor gamma (PPARγ) expression^15^—a result consistent with that of another published work^16^—and that PMQ (10 mg/kg/day e.g.) combats monosodium glutamate (MSG)-induced obesity^17^. PPARγ agonists have been shown to induce mitochondrial biogenesis and a brown adipose phenotype in white adipocytes^18, 19^. Based on these results, we hypothesized that PMQ exerts anti-obesity effects by increasing brown adipogenesis in WAT. To test this hypothesis, we designed a series of experiments involving 3T3-L1 white adipocytes and a high-fat diet (HFD)-induced obesity mouse model.

## Results

### Effects of PMQ on cell viability in 3T3-L1 preadipocytes and adipocytes

**Figure 1:**
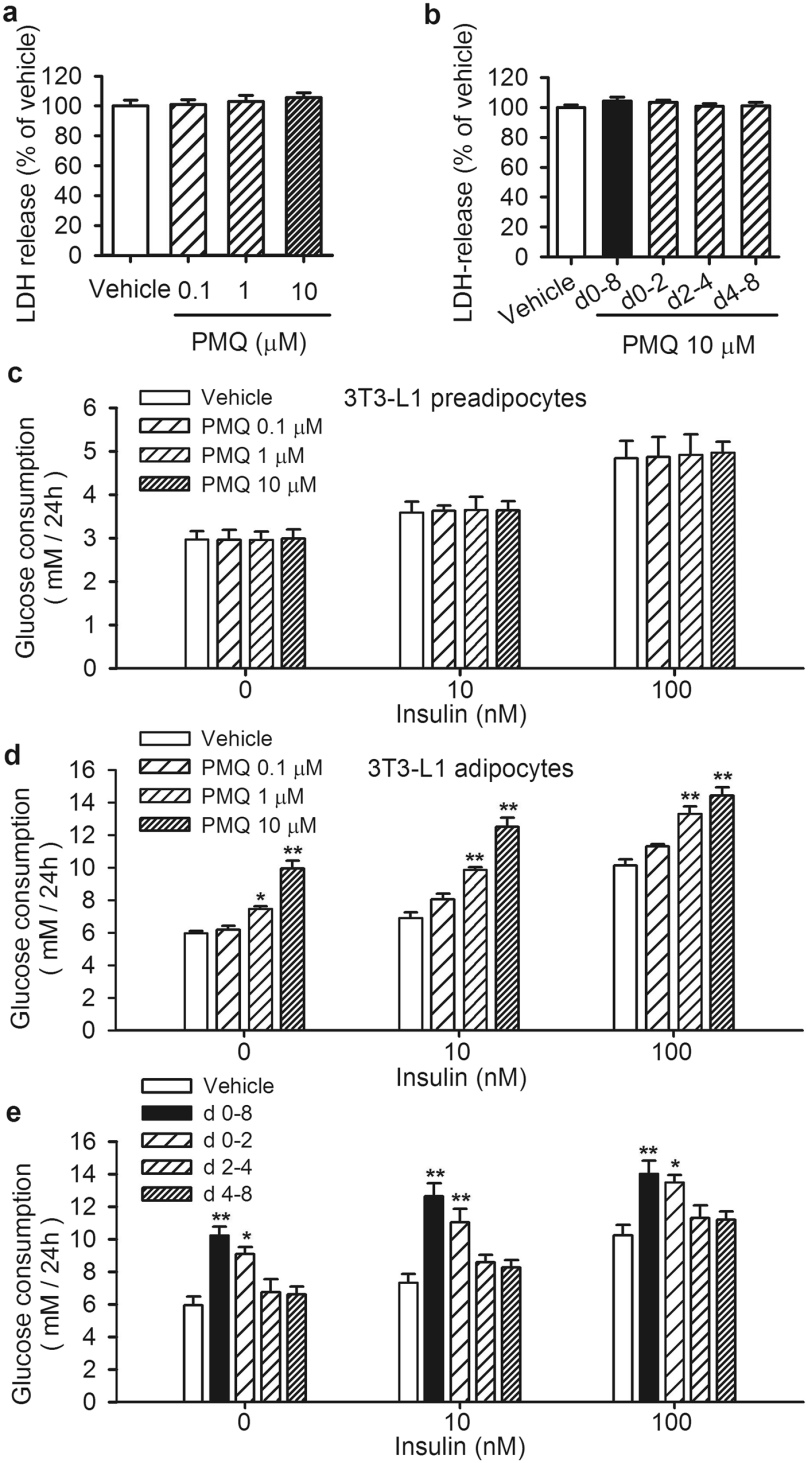
PMQ had no cytotoxic effects and improved glucose metabolism in 3T3-L1 adipocytes. (**a**) 3T3-L1 preadipocytes were treated with PMQ at concentrations of 0.1, 1 and 10 μM for 72 h. (**b**) During the indicated periods (Days 0–8, 0–2, 2–4, 4–8) of 3T3-L1 adipocyte differentiation, the cells were exposed to 10 μM PMQ. Cell toxicity was evaluated by LDH release assay on day 8. (**c**) Effects of 0.1, 1 and 10 μM PMQ on glucose consumption in 3T3-L1 preadipocytes stimulated with or without 10 or 100 nM insulin. (**d**) Cells were incubated with different concentrations of PMQ from Days 0–8. On day 7 of differentiation, the cells with or without 10 or 100 nM insulin were incubated in phenol red-free DMEM with 2% BSA for 24 h. The glucose consumption was determined on day 8. (**e**) Cells were incubated with 10 μM of PMQ during the different exposure periods (Days 0–8, 0–2, 2–4 and 4–8). On day 7 of differentiaion the cells with or without 10 or 100 nM insulin were incubated in phenol red-free DMEM with 2% BSA for 24 h. The glucose consumption was determined on day 8. The data are expressed as the mean ± SEM. *n* = 3 experiments, *p < 0.05, **p < 0.01 vs vehicle.

We investigated the effects of PMQ on cell viability in 3T3-L1 preadipocytes and adipocytes using LDH release and MTT assays. The *in vitro* 3T3-L1 adipocyte differentiation process was divided into the following 3 stages: an early stage (Days 0–2), a middle stage (Days 2–4) and a late stage (Days 4–8). LDH release assay showed that administration of PMQ at concentrations ranging from 0.1 to 10 μM had no cytotoxic effects on 3T3-L1 preadipocytes (Fig. 1a) and that PMQ did not have any cytotoxic effects on 3T3-L1 cells when the cells were exposed to PMQ from Days 0–8, 0–2, 2–4 and 4–8 during the differentiation period (Fig. 1b). MTT assay showed that PMQ did not have an effect on cell proliferation (Fig. S1).

### PMQ improved glucose metabolism in 3T3-L1 adipocytes

To investigate PMQ metabolic activity, we analyzed glucose consumption in 3T3-L1 preadipocytes and adipocytes. We found that PMQ did not increase glucose consumption in 3T3-L1 preadipocytes stimulated with or without insulin (Fig. 1c). In contrast, PMQ increased glucose consumption in a concentration-dependent manner in 3T3-L1 adipocytes stimulated with and without insulin (Fig. 1d). Administration of PMQ at concentrations of 1 and 10 μM increased glucose consumption by 24.6% and 66.4%, respectively, in the absence of insulin (*p* < 0.05 and *p* < 0.01 vs vehicle). We observed synergy between PMQ and insulin, as treatment with 1 and 10 μM PMQ increased insulin-induced glucose consumption by 42.8% and 81.3%, respectively, in the presence of 10 nM insulin (*p* < 0.01 and *p* < 0.01 vs vehicle). These findings suggest that PMQ may promote insulin activity in differentiated 3T3-L1 adipocytes. We subsequently investigated the effects of PMQ on 3T3-L1 adipocyte differentiation during different exposure periods (Days 0–2, Days 2–4 and Days 4–8). As shown in Fig. 1e, PMQ significantly increased glucose consumption in the absence or presence of insulin during Days 0 and 2, which suggests that PMQ promotes glucose metabolism in the early stage of differentiation.

### Effects of PMQ on TG accumulation and lipolysis in 3T3-L1 adipocytes

**Figure 2:**
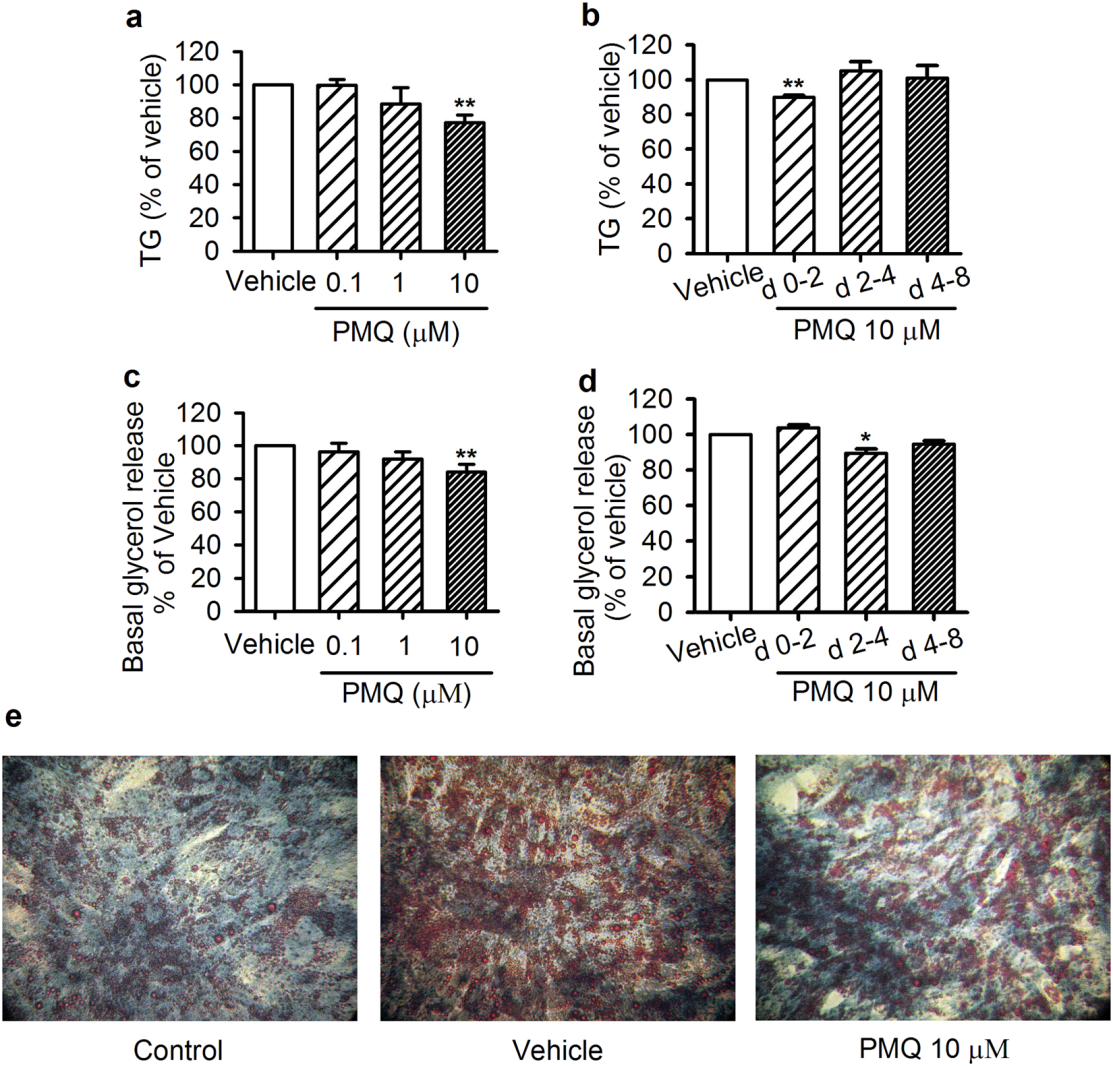
PMQ inhibited TG accumulation and lipolysis in 3T3-L1 adipocytes. (**a**) Cells were incubated with different concentrations of PMQ from Days 0–8. TG accumulation in 3T3-L1 adipocytes was measured on day 8 after the cells had reached confluence. (**b**) Effects of 10 μM PMQ on TG accumulation in 3T3-L1 adipocytes during the different exposure periods (Days 0–8, 0–2, 2–4 and 4–8). The TG accumulation was measured on day 8. (**c**) On day 7 of differentiation, the cells were incubated in phenol red-free DMEM with 2% fatty acid-free BSA for 24 h. Glycerol content in the media was measured. (**d**) Effects of 10 μM PMQ on glycerol release from 3T3-L1 adipocytes during the different exposure periods (Days 0–2, 2–4 and 4–8). Glycerol content in the media was measured on day 8. (**e**) On day 8, preadipocytes (control), adipocytes treated without PMQ (vehicle) and with 10 μM PMQ (PMQ), were fixed, stained with Oil Red O (red, lipid droplets) and Methylene Blue (blue, cytoplasm), and photographed. The data are expressed as the mean ± SEM. *n* = 3 experiments, *p < 0.05, **p < 0.01 vs vehicle.

Lipid storage is an important function of white adipocytes. Administration of 10 μM PMQ significantly decreased intracellular TG content in 3T3-L1 adipocytes (77.1 ± 4.8% of vehicle, *p* < 0.01 vs vehicle, Fig. 2a) during the early stage of differentiation (Days 0–2, Fig. 2b). Oil Red O and Methylene Blue staining (Fig. 2e) showed that treatment with PMQ significantly reduced intracellular red-stained lipid accumulation without decreasing blue-stained adipocyte numbers, consistent with those results of the MTT assay.

WAT lipolysis results in glycerol and nonesterified fatty acid liberation^20^. We found that PMQ administration did not increase glycerol levels in culture supernatants. Rather, 10 μM PMQ significantly inhibited glycerol release from 3T3-L1 adipocytes (84.2 ± 4.6% of vehicle, *p* < 0.01 vs vehicle, Fig. 2c) during the middle stage of differentiation (Days 2–4, Fig. 2d). This finding suggests that PMQ did not increase basal lipolysis.

### Effects of PMQ on adipogenesis or lipogenesis transcription factor and thermogenic-related gene expression in 3T3-L1 adipocytes

**Figure 3:**
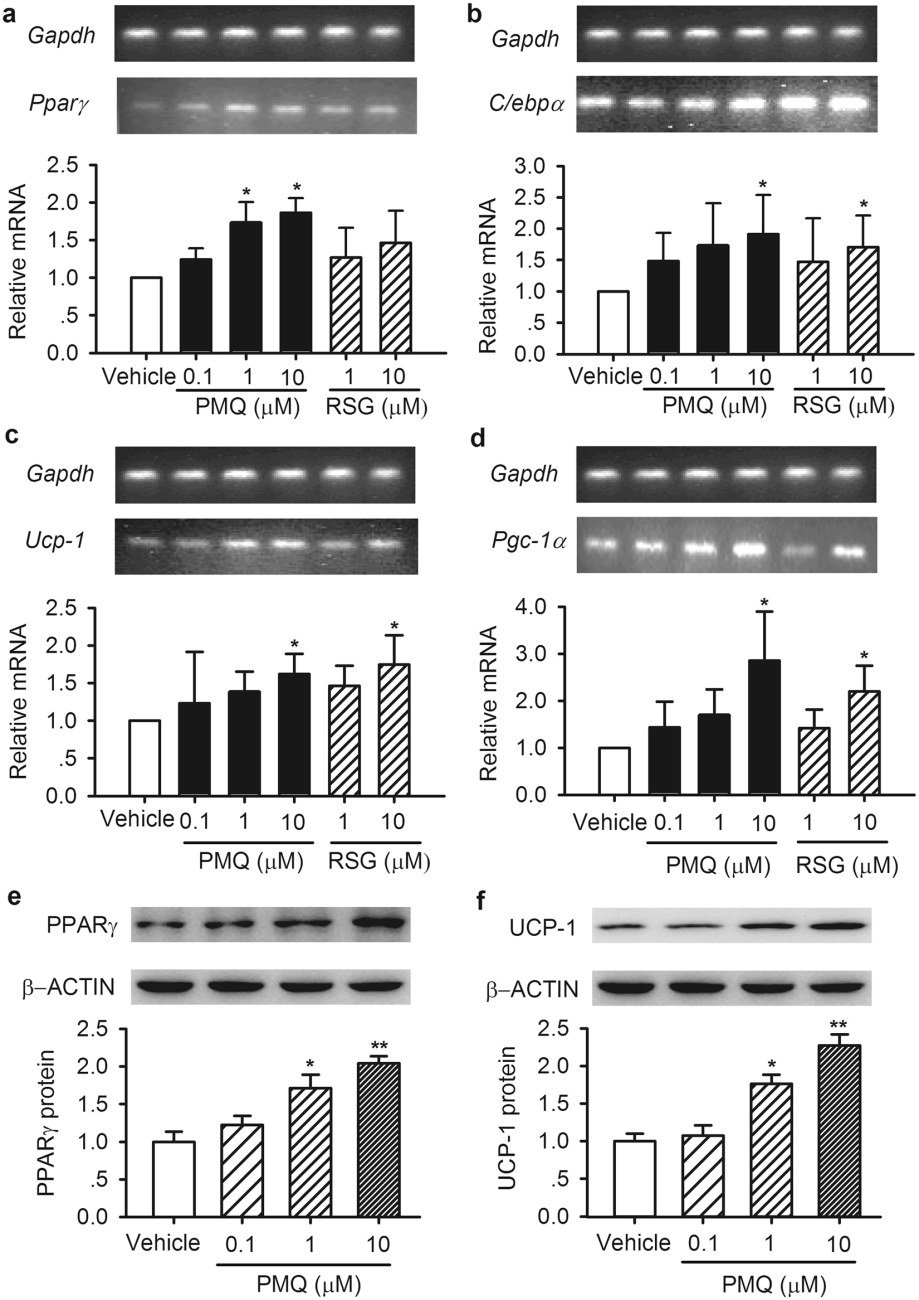
PMQ upregulated the mRNA/protein expression levels of adipogenesis transcription factors and thermogenic-related genes in 3T3-L1 adipocytes. Cells were incubated with different concentrations of PMQ from Days 0–8. (**a–d**) Relative mRNA levels of *Pparγ*, *C/ebpα*, *Ucp-1* and *Pgc-1α* in 3T3-L1 adipocytes treated with or without PMQ. Rosiglitazone-treated cells served as positive controls. (**e,f**) Western blot analysis of PPARγ and UCP-1 protein expression in 3T3-L1 adipocytes treated with the indicated agent. The gels or blots were cropped as indicated. The data are expressed as the mean ± SEM. *n* = 3 experiments, *p < 0.05, **p < 0.01 vs vehicle.

PPARγ and CCAAT/enhancer-binding protein alpha (C/ebpα) are key adipogenesis upregulators. To confirm whether the above mentioned decrease in TG accumulation is caused by downregulation of adipogenesis or lipogenesis transcription factors, such as PPARγ and C/ebpα, we measured the expression levels of related transcription factors in 3T3-L1 adipocytes. We found that 10 μM PMQ upregulated PPARγ and C/ebpα mRNA expression (Fig. 3a,b), as well as glucose transporter type 4 (Glut4) expression (Fig. S2a), and confirmed that 10 μM PMQ also upregulated PPARγ protein expression (Fig. 3e) in 3T3-L1 adipocytes. These results suggest that PMQ did not inhibit TG accumulation through PPARγ and C/ebpα downregulation in 3T3-L1 adipocytes.

UCP-1 is highly expressed in brown adipocytes and is recognized as a marker of those cells^21^. Ucp-1 mRNA and protein expression levels in 3T3-L1 adipocytes were significantly elevated by treatment with 10 μM PMQ (Fig. 3c,f). We also found that 10 μM PMQ could upregulate the mRNA expression of peroxisome proliferator-activated receptor gamma, coactivator 1 alpha (Pgc-1α, Fig. 3d), and other thermogenic-related genes, such as cell death-inducing DNA fragmentation factor, alpha subunit-like effector A (Cidea), bone morphogenetic protein 7 (Bmp7) and PR domain containing 16 (Prdm16) (Fig. S2a). These results demonstrate that PMQ can convert 3T3-L1 adipocytes (classical white adipocytes) to “brown” adipocytes.

**Figure 4:**
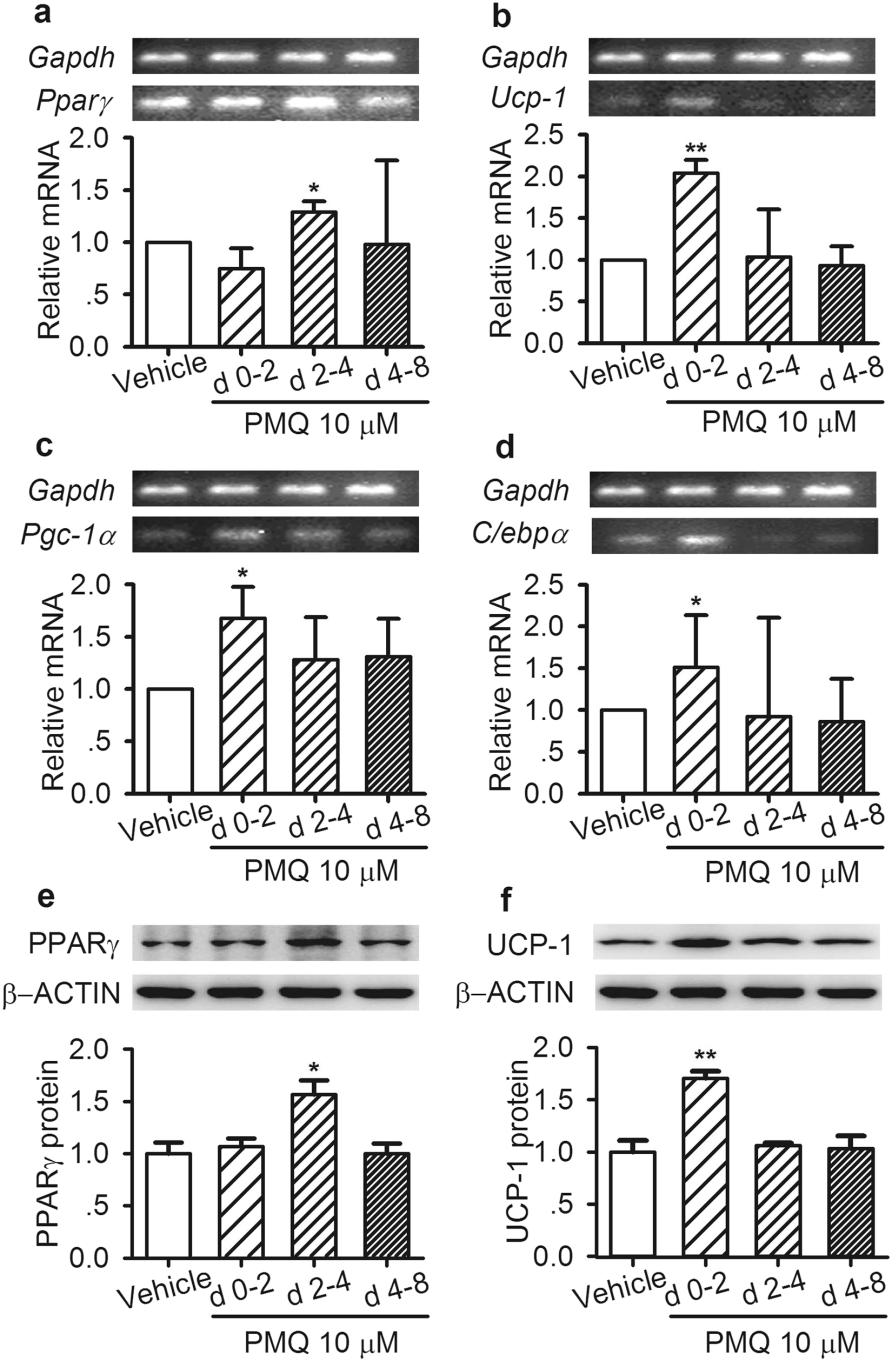
PMQ affected the expression of adipogenesis-related transcription factors and thermogenic-related genes in 3T3-L1 adipocytes during different differentiation stages. Cells were incubated with 10 μM PMQ during the indicated periods, Days 0–2, 2–4 and 4–8. (**a–d**) On day 8, the relative mRNA levels of *Pparγ*, *Ucp-1*, *Pgc-1α and C/ebpα* in 3T3-L1 adipocytes treated with or without PMQ. (**e,f**) On day 8, western blot analysis of PPARγ and UCP-1 protein expression in 3T3-L1 adipocytes treated with the indicated agents. The gels or blots were cropped as indicated. The data are expressed as the mean ± SEM. *n* = 3 experiments; *p < 0.05, **p < 0.01 vs vehicle.

We subsequently investigated how PMQ affected the expression of the above factors or genes in 3T3-L1 adipocytes during different differentiation stages (Days 0–2, Days 2–4 and Days 4–8). We found that PMQ increased PPARγ mRNA and protein expression levels during the middle stage of differentiation (Days 2–4) (Fig. 4a,e) and upregulated Ucp-1 expression during the early stage of differentiation (Days 0–2) (Fig. 4b,f). PMQ treatment also increased Pgc-1α (Fig. 4c), C/ebpα (Fig. 4d) and Prdm16 (Fig. S2b) mRNA expression levels during the early stage of differentiation (Days 0–2).

### Anti-obesity effects of PMQ in HFD mice

**Figure 5:**
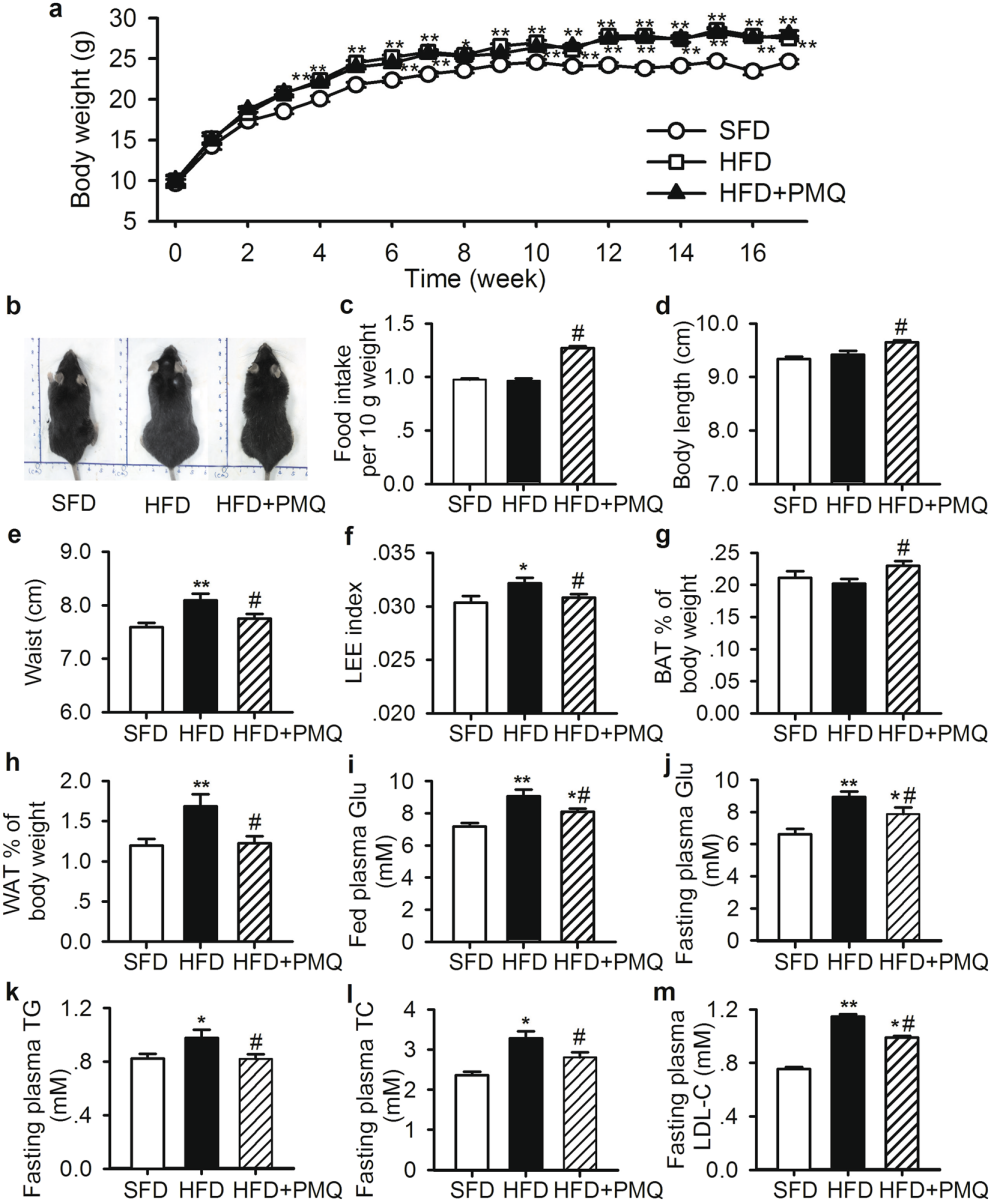
PMQ protected mice against high fat diet-induced obesity. (**a**) Body weights, (**b**) representative images, (**c**) food intake, (**d**) body length, (**e**) waist circumference, (**f**) LEE index, (**g,h**) interscapular BAT and epididymal WAT weight, (**i**) fed plasma glucose levels, (**j**) fasting plasma glucose levels, (**k**) TG levels, (**l**) TC levels, and (**m**) LDL-C levels of HFD mice. The data are expressed as the mean ± SEM. *n* = 10 experiments, *p < 0.05 **p < 0.01 vs SFD; ^*#*^ *p* < *0*.05 vs HFD.

The effects of PMQ on HFD-induced obesity *in vivo* were investigated. Representative images of the mice are shown in Fig. 5b. The weight gains in the standard-fat diet (SFD) and HFD control groups during the 17-week period were 15.0 ± 0.3 g and 17.9 ± 0.5 g (*p* < 0.05), respectively. At the end of experiment, we found that PMQ did not significantly lower the body weight in PMQ-treated mice compared with HFD control mice (27.5 ± 0.29 g vs 27.9 ± 0.49 g, Fig. 5a); however, PMQ did increase food intake in these mice compared with HFD control mice (Fig. 5c, *p* < 0.05 vs HFD control group). Thus, feeding efficiency, which was calculated by dividing weight gain by total food intake, was reduced in the PMQ-treated group compared with the HFD control group. Consistent with these results, the body length was increased (Fig. 5d), and the waist circumference was reduced (Fig. 5e) in the PMQ-treated group compared with the HFD control group. The Lee index was also significantly reduced in PMQ-treated mice compared with HFD control mice (Fig. 5f). Interscapular BAT weight was increased by 13.7 ± 3.5% (Fig. 5g, *p* < 0.05 vs HFD control group) in the PMQ-treated group compared with the HFD control group. The epididymal WAT weight of the HFD control group was increased by 41.7 ± 2.8% (1.69 ± 0.15% of body weight, *p* < 0.01) compared with that of the SFD group (1.20 ± 0.08% of body weight), while the epididymal WAT weight of the PMQ-treated group was decreased compared with that of the HFD control group (1.22 ± 0.09% of body weight, *p* < 0.05 vs HFD control group, Fig. 5h).

### PMQ improved glucose and lipid metabolism in HFD mice

As shown in Fig. 5i–m, HFD mice displayed significantly higher plasma glucose (fed or fasting), total cholesterol (TC), TG and low-density lipoprotein cholesterol (LDL-C) levels than SFD mice (*p* < 0.05 *or p* < 0.01 vs SFD group). PMQ administration resulted in significant reductions in serum glucose, TC, TG and LDL-C levels in PMQ-treated mice compared with HFD control mice (*p* < 0.05 vs HFD control group).

### Morphological and molecular changes induced by PMQ in adipose tissue

**Figure 6:**
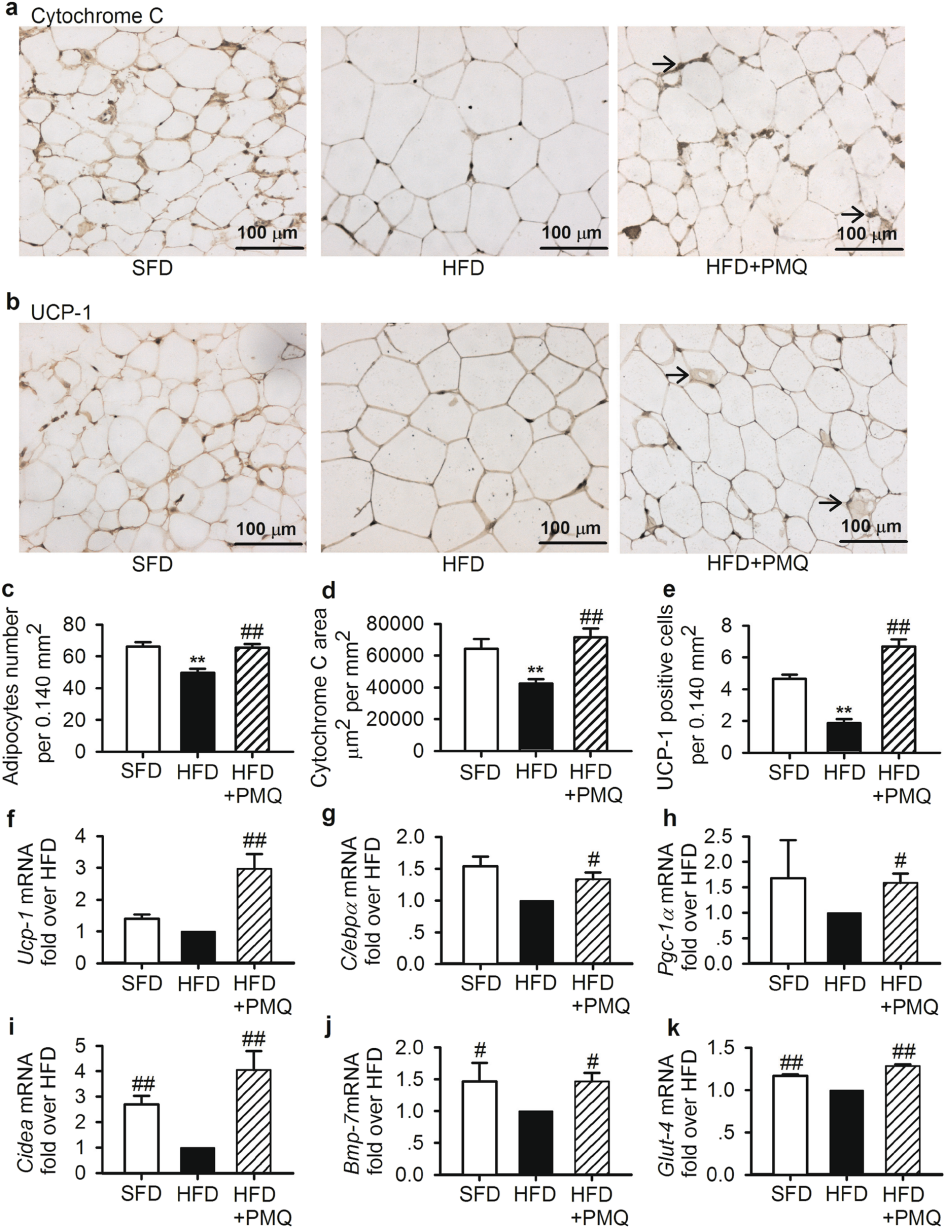
PMQ induced morphological and molecular changes in adipose tissue in HFD mice. Epididymal adipose tissues from HFD mice treated with the indicated agents (n = 10 experiments) were stained with mitochondrial markers. (**a**,**b**) Representative images of cytochrome C and UCP-1 staining. The arrows indicate cytochrome C-enriched or UCP1-enriched brown adipocytes. (**c**) Morphometric analysis of adipocyte size, which was determined based on the average numbers of cells in the acquired images (total area = 0.14 mm^2^). (**d**) Morphometric analysis of cytochrome C-stained areas. (**e**) Average numbers of UCP1-positive adipocytes in the acquired images (total area = 0.14 mm^2^). (**f–k**) qPCR analysis of the mRNA levels of the following brown fat-selective genes in epididymal fat tissue: *Ucp-1*, *C/ebpα*, *Pgc-1α*, *Cidea*, *Bmp7*, and *Glut-4* (*n* = 5 experiments). The data are expressed as the mean ± SEM. **p < 0.01 vs SFD; ^*#*^ *p* < *0.05*, ^*##*^ *p* < 0.01 vs HFD.

Given our above mentioned observations regarding the effects of PMQ treatment, we examined whether PMQ induces browning of white adipose tissues in HFD mice. H&E staining of epididymal adipose tissue showed that adipocyte size in HFD mice was generally larger than that in SFD mice. Adipocyte size was smaller in PMQ-treated mice than in HFD control mice, as the number of adipocytes per 0.144 mm^2^ was increased by 31.7 ± 4.6% in the former group compared with the latter group (*p* < 0.01 vs HFD control mice, Fig. 6c). Mitochondria play a crucial role in cellular energy generation. Immunohistochemical staining showed that the expression of cytochrome C, a mitochondrial marker, was significantly increased in PMQ-treated mice compared with HFD control mice (Fig. 6a,d), indicating that mitochondria-poor adipocytes had been converted to mitochondria-rich adipocytes. Induction of uncoupling protein expression is associated with the acquisitioning of brown fat features. Treatment with PMQ increased the numbers of UCP1-positive stained multilocular cells in PMQ-treated mice compared with HFD control mice, as shown in Fig. 6b,e. Consistent with the above results pertaining to cell morphological changes, the qPCR results showed that the expression of the Ucp1 gene, as well as that of other thermogenic-related genes, including C/ebpα, Pgc-1α, Cidea, Bmp7 and Glut-4, was significantly upregulated in the epididymal adipose tissue of PMQ-treated mice (Fig. 6f) compared with that of HFD mice (Fig. 6g–k).

## Discussion

PMQ is a flavonoid compound and has been shown to have great anti-diabetic effects on streptozotocin-induced diabetic rats^22^ and anti-obesity effects on MSG mice^17^. In this study, we found that PMQ could attenuate TG accumulation in 3T3-L1 adipocytes, combat hyperglycemia and hyperlipidemia and attenuate HFD-induced increases in the epididymal WAT mass in C57BL/6 mice.

Initially, we attributed the above findings to the possible cytotoxic effects of PMQ as its homolog, quercetin, on fat cells^23, 24^; however, we found that treatment with 10 μM PMQ suppressed intracellular lipid accumulation without exerting cytotoxic effects or inhibiting *Pparγ* and *C/ebpα* expression. These findings suggest that PMQ exerts anti-obesity effects through a unique mechanism.

Many anti-obesity strategies have been used against white adipocytes, including strategies involving drugs targeting adipogenesis, lipogenesis and lipolysis and energy expenditure. Thus, in addition to evaluating PMQ-induced cytotoxicity, we analyzed the effects of PMQ on adipogenesis, lipogenesis, lipolysis, glucose utilization, and energy expenditure. In adipocytes, PMQ could upregulate the expression of the most important adipogenesis and lipogenesis factor, PPARγ; increase the consumption of glucose; and increase the expression of the glucose regulators *C/ebpα* and *Glut-4*. We therefore surmised that, theoretically, PMQ should not decrease cell lipid storage. We also showed that PMQ inhibited lipolysis. Consistent with this result, we showed that PMQ inhibited the expression of TNFα, a virtual regulator of lipolysis, in another study^15^. PMQ elevated carnitine palmitoyltransferase 1 (*Cpt-1*) mRNA expression levels (although not significantly, Fig. S2a) in 3T3-L1 adipocytes, thereby improving fatty acid oxidation. Similar results were observed in studies involving cardiomyocytes^25^ and myocytes^17^. PMQ also upregulated *Pgc-1α* and *Ucp-1* gene expression and increased mitochondrial biogenesis, changes associated with increases in energy expenditure. Because UCP-1 is a predominant marker of brown adipocytes, we surmised that PMQ may turn “white” fat cells into “brown” ones. The expression of other brown genes that are barely expressed in white adipocytes, such as *Cidea*, *Prdm16*, *Bmp7*, was also demonstrated to be upregulated by PMQ in 3T3-L1 adipocytes and the epididymal fat of HFD mice. PMQ also significantly reduced feeding efficiency, epididymal fat weights and serum lipid levels and increased interscapular brown fat pad sizes. These findings suggested that a relationship exists between the anti-obesity effects of PMQ and energy expenditure.

In our previous study, we found that PMQ had a significant weight loss inducing-effect in MSG mice^17^. The MSG mouse model is an obesity model induced by MSG administration during the neonatal period that presents as multiple metabolic disorders. Food restriction is less efficient in MSG-induced obesity animals than in other animals^26^. In contrast, the HFD mouse model is a diet-induced obesity animal model. HFD mice consumed more calories than SFD mice. Food restriction is very efficient in HFD-induced obesity animals^27^. In this study, HFD mice treated with PMQ consumed much more food than HFD control mice, but their body weight gain was unchanged compared with that of their counterparts, partly because PMQ enhances energy expenditure. In our previous study we also found that PMQ upregulated PGC-1α and PPARα expression in MSG-treated mice^17^. Whether PMQ has browning effects in MSG mice will be investigated in future studies.

We also investigated the mechanism underlying the “browning” effects of PMQ on white fat cells. PPARγ plays the central role in the differentiation of both white and brown fat cells, and is considered a regulator of the differentiation-dependent expression of UCP-1^28, 29^. Thiazolidinediones activate PPARγ and induce UCP-1 expression in white adipocytes^19^. However, in the brown adipocytes differentiation process, UCP-1 expression augmentation occurs in the setting of no upregulation or downregulation of PPARγ gene expression, which suggests that UCP-1 gene expression is not a simple effect of the presence of this protein^30^. Ectopic expression of PPARγ in mesenchymal cells induces a white fat, but not a brown fat phenotype, suggesting that this molecule alone does not determine brown fat cell fates^31^. Our results showed that PMQ activated PPARγ and induced UCP-1 expression in 3T3-L1 adipocytes. However, PMQ increased PPARγ expression and lipolysis in the absence of UCP-1 expression augmentation and intracellular TG inhibition during PMQ treatment in the middle stage of 3T3-L1 differentiation, indicating that PPARγ may be involved indirectly in the process of PMQ-induced browning.

PGC-1α was first identified as a PPARγ-interacting protein from brown fat cells and is recognized as the predominant regulator of mitochondrial biogenesis and oxidative metabolic pathways in many cell types, wherein it coactivates various transcription factors. Ectopic expression of PGC-1α in white fat cells induces the expression of several mitochondrial genes and thermogenic genes, including *Ucp-1* ^32^. A recent study identified a *Pgc-1α*-dependent myokine that drives brown-fat-like development of white fat and thermogenesis^33^. Flavonoids, such as quercetin and polymethyloxyisoflavones, can increase *Pgc-1α* expression and mitochondrial biogenesis in the brain, muscle and renal proximal tubular cells^34, 35^. Both these reports and our previous study^17^ showed that PMQ can upregulate *Pgc-1α* expression in adipocytes and myocytes. PRDM16 was identified as one of only three genes whose expression correlated strongly with the brown fat phenotype *in vivo* and in cultured cell models^36^. BMP7 singularly promotes the differentiation of brown preadipocytes and induces the early regulators of brown fat fate, PRDM16 and PGC-1α^37^. When ectopically expressed in cultured mesenchymal cells, including white fat preadipocytes, both PRDM16 and BMP7 can induce a complete brown fat differentiation process involving the activation of thermogenic genes (UCP-1, PGC-1α), mitochondrial genes and other BAT-selective genes (*Cidea*)^31, 36–39^. PMQ may enhance energy expenditure as a BMP7 and PRDM16 upregulator in adipocytes.

PMQ can also be used to treat metabolic disease to improve glucose tolerance and insulin sensitivity in MSG-induced obese mice^17^ and diabetic rats^22^ through a process unrelated to insulin release. We found PMQ can increase *Glut-4* and *Cebp/α* expression in 3T3-L1 adipocytes and the epididymal fat of HFD mice, thereby augmenting glucose consumption in fat cells, a change that may reduce serum glucose and lipid levels and appears to be at least partially responsible for the anti-metabolic disorder effects of this chemical.

Although PMQ may induce browning of WAT, the mechanisms of action of PMQ on fat depots in mice remains unknown. AMP-activated protein kinase (AMPK) has been proved to directly phosphorylate PGC-1α protein and increase PGC-1α-dependent activation of its own promoter in skeletal muscle^40^. Recent studies showed that a PGC-1α-dependent myokine termed irisin, which is proteolytically cleaved from fibronectin-type III domain-containing 5 (FNDC5) in muscle, could stimulate thermogenesis and browning of WAT both in cultured adipocytes and *in vivo* ^33, 41^. Intriguingly, our previous study showed that in addition to activating AMPK of gastrocnemius muscle in MSG-induced obese mice, PMQ treatment could activate AMPK and elevate PGC-1α expression in C2C12 myotube^17^. Therefore, it would be worth investigating further whether the AMPK-PGC-1α-FNDC5 pathway in skeletal muscle is involved in the action of PMQ on fat depots in mice.

It should be noted that in current study, 3T3-L1 cells were induced to differentiate only with 10 μg/ml insulin. Insulin markedly increases the rates of triglyceride synthesis and accumulation in 3T3-L1 cells^42^ and induces more than 75% of these cells to differentiate into adipocytes^43^. We actually also tested the effects of PMQ with a differentiation cocktail (10 μg/ml insulin, 0.5 μM isobutylmethylxanthine and 1 μM dexamethasone) and PMQ did not show any beneficial effects on 3T3-L1 cells. We could not explain why the results were not consistent with those observed in 3T3-L1 adipocytes induced only with insulin. One of possibilities for the discrepancy is likely related to the influence of isobutylmethylxanthine and/or dexamethasone on the effect of PMQ, which should be investigated in detail by making comparative study in the future.

The present study had some limitations. One of limitations was that we did not perform the calorie excretion measurement. Feeding efficiency was calculated by dividing weight gain by total food intake. Although we did find that the food consumption of PMQ group increased gradually and no abnormal defecation was observed, it is possible that mice fed PMQ diet had significantly higher or lower excretion which would change the feeding efficiency. The calorie excretion measurement will be performed in the future to rule out the possibility. Another limitation was that we only investigated the effects of PMQ on 3T3-L1 preadipocytes, whether PMQ can also modify cell fate during *in vitro* differentiation of primary mouse white preadipocytes remains to be studied.

In summary, our *in vitro* and *in vivo* data show that PMQ can enhance the expression of genes such as Ucp-1, Cidea, Pgc-1α, Prdm16, Bmp7, Glut4, C/ebpα in white adipocytes. PMQ also exerts beneficial effects on glucose and lipid metabolism in HFD mice. The browning effects of PMQ are associated with increases in both energy expenditure and glucose tolerance in HFD mice. Our findings suggest that PMQ may induce browning of adipose tissue, a phenomenon that is at least partly related to its anti-obesity effects. PMQ may be a potential candidate drug for the treatment of obesity.

## Methods

### Cell culture and cell viability measurement

Mouse 3T3-L1 preadipocytes obtained from the American Type Culture Collection (Manassas, VA, USA) were incubated in Dulbecco’s modified Eagle’s medium (DMEM) containing 10% fetal bovine serum, 100 U/ml penicillin and 100 mg/ml streptomycin at 37 °C in a humidified atmosphere of 5% CO_2_. Two days after the preadipocytes reached confluence (designated Day 0) we cultured them in differentiation medium (DM, 10% FBS, 10 μg/ml insulin) for 4 days to induce differentiation and then transitioned them to 10% FBS DMEM, in which we cultured them for an additional 4 days^43^. PMQ, which was synthesized at a purity of 99.5% by the Food & Drug Evaluation Centre of Tongji Medical College at Huazhong University of Science and Technology, as determined by HPLC^15^, was dissolved in DMSO when added to the medium. 3T3-L1 preadipocytes cultured in 96-well culture plates were treated with DMSO or PMQ at a concentration of 0.1, 1 or 10 μM for 72 h. In the stage-specific experiments, 3T3-L1 adipocytes undergoing differentiation were exposed to 10 μM PMQ during Days 0–8, 0–2, 2–4 and 4–8, respectively, while in the vehicle control group, the cells were treated with DMSO for 8 days (Supplementary Table S1).

MTT analysis was performed as described previously^15^. After pretreating the cells with PMQ, we added 20 μl of MTT (Amresco, Solon, USA; 5 mg/ml) to each well and incubated the cells for 4 h, after which we measured the absorbance at 490 nm using a microplate reader. The lactate dehydrogenase (LDH) concentration in the medium was detected with an LDH Assay Kit (Beyotime Inc., China), according to the manufacturer’s instructions.

### Glucose consumption assay

As described previously^44^, we performed the glucose consumption test using colorimetric assay kits (BioSino, Beijing, China). 3T3-L1 cells were grown in 96-well plates. Similar to the MTT assay, in this assay, the cells were treated with different concentrations of PMQ at different times. Upon performing the glucose consumption test, we replaced the normal culture medium with phenol red-free DMEM supplemented with 0.25% bovine serum albumin (BSA) containing insulin (final concentration 0, 10, 100 nM). After the cells had incubated for 24 h, we determined the glucose concentration in the medium using colorimetric assay kits, in accordance with the manufacturer’s instructions. The amount of glucose consumed was obtained by subtracting the glucose concentration of the wells containing cells from the glucose concentration of the blank wells.

### Measurement of triglyceride concentrations

We lysed differentiated 3T3-L1 adipocytes in PBS containing 1% Nonidet P-40 and analyzed whole-cell lysates to determine TG content using a TG (GPO) Reagent Set (BioSino). The results were normalized by the total protein in the cell layer using an Enhanced BCA Protein Assay Kit (Beyotime, Jiangsu, China).

### Lipolysis assay

Differentiated 3T3-L1 adipocytes were incubated in phenol red–free DMEM with 2% fatty acid–free BSA for 24 h at 37 °C. The glycerol content in the media was measured colorimetrically against a set of glycerol standards using a Glycerol Determination (GPO-POD) Kit (Chaoyan, Shanghai, China).

### Oil Red O staining

Oil Red O staining was performed on day 8. The cells were washed with phosphate-buffered saline twice, fixed with 10% formalin for 1 h, and then stained with Oil Red O for 20 min. The cells were subsequently photographed with or without Methylene Blue staining.

### Animal treatment and experimental protocol

All animal experiments were approved by the Ethics Committee of Animal Use for Teaching and Research of Tongji Medical College at Huazhong University of Science and Technology, and all experimental procedures were conducted in accordance with the Guide for the Care and Use of Laboratory Animals, which was published by the US National Institutes of Health (NIH Publication No. 85-23, revised 1996). Healthy C57BL/6 mice (4–5 weeks) were purchased from the Center of Experimental Animals (Wuhan University, China). These animals were maintained in a temperature- and humidity-regulated room (22 ± 2 °C, 55 ± 5%, respectively) under a 12-h light/dark cycle. The mice were randomly divided into the following three groups: a group fed a standard-fat diet (SFD, 7.7% total energy from fat), a group fed a mild HFD (27.6% total energy from fat), and a treatment group fed an HFD plus 0.04% (g/g) PMQ (HFD + PMQ group). After receiving the above diets for 17 weeks, the mice were anesthetized with diethyl ether, and blood samples were taken from their inner canthi to measure fed plasma glucose levels. Over a fortnight, blood samples were collected after 6-h fasting periods, and the serum was separated from each of the samples. All the mice were then killed, and their epididymal white fat pads and interscapular brown fat pads were removed, weighed and frozen at −80 °C.

### Serum chemistry analysis

Serum glucose, TG, TC and LDL-C levels were measured using colorimetric assay kits (BioSino), and insulin levels were measured with a double-antibody radioimmunoassay kit (North Institute of Biological Technology, Beijing, China).

### Histological analysis

The epididymal fat pads were removed, formalin-fixed, paraffin-embedded and sectioned for cytochrome C (Santa Cruz, CA, USA) and UCP1 (Santa Cruz, CA, USA) immunohistochemical staining. The cytochrome C-positive areas, UCP 1-positive cell numbers and cell populations were quantified using an image analysis program (Image-Pro Plus 6.0), as previously described^18^.

### RT-PCR and quantitative real-time PCR (qPCR)

Total RNA was extracted from the 3T3-L1 cells and epididymal adipose tissue specimens using Trizol reagent (Invitrogen), according to the manufacturer’s instruction. Reverse transcription (RT) was performed in a 50-μl reaction mixture using oligo18 primers, in accordance with an RT system protocol^31^. After the RT procedure, the reaction mixture (cDNA) was used for PCR and qPCR.

The primers for the corresponding genes that were used for RT-PCR are shown in Supplementary Table S1. The reaction comprised the following steps: an initial denaturation of the template for 5 minutes at 94 °C, followed by 40 repeating cycles of denaturing for 30 seconds at 50–63 °C, annealing for 30 seconds, extension for 1 minute at 72 °C, and a final elongation at 72 °C for 10 minutes. The PCR products were size-fractionated by 2.5% agarose gel electrophoresis and visualized by SYBR Green I (Solarbio) staining.

The sequences of the qRCR primers used in the study are listed in Supplementary Table S2. cDNA was subjected to qPCR (10 ng of cDNA per reaction), as described previously^18^. PCR was performed with Universal PCR Master Mix 2X (Applied Biosystems) using a 7900HT Sequence Detector System (Applied Biosystems), as recommended by the vendor. The relative expression levels of the target gene RNA were normalized to those of the housekeeping gene β-actin and were expressed as fold changes.

### Western blotting

After PMQ treatment, the 3T3-L1 cells were washed with PBS (1×) thrice and lysed on ice in RIPA buffer 30 min. The supernatants were collected from the lysates after the cells were centrifuged (12000 g, 15 min), and the protein concentrations were subsequently determined using an Enhanced BCA Protein Assay Kit (Beyotime, Jinagsu, China). Equal amounts of protein were separated by 10% SDS-polyacrylamide gel electrophoresis (PAGE), transferred to a polyvinylidene difluoride membrane (Millipore, MA, USA), and hybridized with primary antibodies specific for UCP-1 (1:800, Abcam), PPARγ (1:600, Proteintech), and β-actin (1:6000, CST) overnight at 4 °C. The membranes were then probed with the appropriate horseradish peroxidase-conjugated secondary antibodies (1:6000, Boster, China) for 1 h at room temperature. Finally, the protein bands were visualized with an enhanced chemiluminescent (ECL) system and quantified by densitometric analysis.

### Statistical analysis

The results are presented as the mean ± SEM. Comparisons between groups were made with ANOVA, and significance was assessed by Tukey’s test. *p* < 0.05 was considered statistically significant.

## Electronic supplementary material

Effects of PMQ on 3T3-L1 adipocytes and mouse gene-specific primers for PCR

## References

[1] Smith KB, Smith MS (2016). Obesity statistics. Prim. Care.

[2] Abente EJ, Subramanian M, Ramachandran V, Najafi-Shoushtari SH (2016). MicroRNAs in obesity-associated disorders. Arch. Biochem. Biophys..

[3] Daneschvar HL, Aronson MD, Smetana GW (2016). FDA approved anti-obesity drugs in the United States. Am. J. Med..

[4] Cypess AM (2009). Identification and importance of brown adipose tissue in adult humans. N. Engl. J. Med..

[5] Nicholls DG, Locke RM (1984). Thermogenic mechanisms in brown fat. Physiol. Rev..

[6] Cannon B, Nedergaard J (2004). Brown adipose tissue: Function and physiological significance. Physiol. Rev..

[7] Gesta S, Tseng YH, Kahn CR (2007). Developmental origin of fat: tracking obesity to its source. Cell.

[8] Celi FS (2009). Brown adipose tissue–when it pays to be inefficient. N. Engl. J. Med..

[9] Ghorbani M, Himms-Hagen J (1997). Appearance of brown adipocytes in white adipose tissue during CL 316,243-induced reversal of obesity and diabetes in Zucker fa/fa rats. Int. J. Obes. Relat. Metab. Disord..

[10] Kopecky J, Clarke G, Enerback S, Spiegelman B, Kozak LP (1995). Expression of the mitochondrial uncoupling protein gene from the aP2 gene promoter prevents genetic obesity. J. Clin. Invest..

[11] Virtanen KA (2009). Functional brown adipose tissue in healthy adults. N Engl J Med.

[12] Hibasami H (2005). Isolation of five types of flavonol from seabuckthorn (Hippophae rhamnoides) and induction of apoptosis by some of the flavonols in human promyelotic leukemia HL-60 cells. Int. J. Mol. Med..

[13] Zhang XH, Liu ZM, He T, Yang F, Jin MW (2008). Effects of pentamethylquercetin on contractility and electrophysiology of cardiac muscle in guinea pigs. Acta Medicinae Universitatis Science of Technologiae Huazhong.

[14] Mao Z, Liang Y, Du X, Sun Z (2009). 3,3′,4′,5,7-pentamethylquercetin reduces angiotensin II-induced cardiac hypertrophy and apoptosis in rats. Can. J. Physiol. Pharmacol..

[15] Chen L (2011). Pentamethylquercetin improves adiponectin expression in differentiated 3T3-L1 cells via a mechanism that implicates PPARgamma together with TNF-alpha and IL-6. Molecules.

[16] Matsuda H (2011). Structural requirements of flavonoids for the adipogenesis of 3T3-L1 cells. Bioorg. Med. Chem..

[17] Shen JZ (2012). Pentamethylquercetin generates beneficial effects in monosodium glutamate-induced obese mice and C2C12 myotubes by activating AMP-activated protein kinase. Diabetologia.

[18] Rong JX (2007). Adipose mitochondrial biogenesis is suppressed in db/db and high-fat diet-fed mice and improved by rosiglitazone. Diabetes.

[19] Vernochet C (2009). C/EBPalpha and the corepressors CtBP1 and CtBP2 regulate repression of select visceral white adipose genes during induction of the brown phenotype in white adipocytes by peroxisome proliferator-activated receptor gamma agonists. Mol. Cell. Biol..

[20] Duncan RE, Ahmadian M, Jaworski K, Sarkadi-Nagy E, Sul HS (2007). Regulation of lipolysis in adipocytes. Annu. Rev. Nutr..

[21] Ricquier D, Bouillaud F (2000). The uncoupling protein homologues: UCP1, UCP2, UCP3, StUCP and AtUCP. Biochem. J..

[22] Wang Y (2011). Anti-diabetic effects of pentamethylquercetin in neonatally streptozotocin-induced diabetic rats. Eur. J. Pharmacol..

[23] Yang JY (2008). Enhanced inhibition of adipogenesis and induction of apoptosis in 3T3-L1 adipocytes with combinations of resveratrol and quercetin. Life Sci..

[24] Ahn J, Lee H, Kim S, Park J, Ha T (2008). The anti-obesity effect of quercetin is mediated by the AMPK and MAPK signaling pathways. Biochem. Biophys. Res. Commun..

[25] He T (2012). *In vivo* and *in vitro* protective effects of pentamethylquercetin on cardiac hypertrophy. Cardiovasc. Drugs Ther..

[26] Luz J (2010). Effect of food restriction on energy expenditure of monosodium glutamate-induced obese rats. Ann Nutr Metab.

[27] Duncan MJ (2016). Restricting feeding to the active phase in middle-aged mice attenuates adverse metabolic effects of a high-fat diet. Physiol Behav.

[28] Sears IB, MacGinnitie MA, Kovacs LG, Graves RA (1996). Differentiation-dependent expression of the brown adipocyte uncoupling protein gene: regulation by peroxisome proliferator-activated receptor gamma. Mol Cell Biol.

[29] Villarroya F, Iglesias R, Giralt M (2007). PPARs in the Control of Uncoupling Proteins Gene Expression. PPAR Res.

[30] Nedergaard J, Petrovic N, Lindgren EM, Jacobsson A, Cannon B (2005). PPARgamma in the control of brown adipocyte differentiation. Biochim. Biophys. Acta..

[31] Ahfeldt T (2012). Programming human pluripotent stem cells into white and brown adipocytes. Nat. Cell Biol..

[32] Tiraby C, Langin D (2003). Conversion from white to brown adipocytes: A strategy for the control of fat mass?. Trends Endocrinol. Metab..

[33] Bostrom P (2012). A PGC1-alpha-dependent myokine that drives brown-fat-like development of white fat and thermogenesis. Nature.

[34] Rasbach KA, Schnellmann RG (2008). Isoflavones promote mitochondrial biogenesis. J. Pharmacol. Exp. Ther..

[35] Davis JM, Murphy EA, Carmichael MD, Davis B (2009). Quercetin increases brain and muscle mitochondrial biogenesis and exercise tolerance. Am. J. Physiol. Regul. Integr. Comp. Physiol.

[36] Seale P (2007). Transcriptional control of brown fat determination by PRDM16. Cell Metab..

[37] Tseng YH (2008). New role of bone morphogenetic protein 7 in brown adipogenesis and energy expenditure. Nature.

[38] Seale P (2008). PRDM16 controls a brown fat/skeletal muscle switch. Nature.

[39] Kajimura S (2008). Regulation of the brown and white fat gene programs through a PRDM16/CtBP transcriptional complex. Genes Dev..

[40] Jager S, Handschin C, St-Pierre J, Spiegelman BM (2007). AMP-activated protein kinase (AMPK) action in skeletal muscle via direct phosphorylation of PGC-1alpha. Proc Natl Acad Sci USA.

[41] Zhang Y (2014). Irisin stimulates browning of white adipocytes through mitogen-activated protein kinase p38 MAP kinase and ERK MAP kinase signaling. Diabetes.

[42] Green H, Kehinde O (1975). An established preadipose cell line and its differentiation in culture. II. Factors affecting the adipose conversion. Cell.

[43] Reed BC, Kaufmann SH, Mackall JC, Student AK, Lane MD (1977). Alterations in insulin binding accompanying differentiation of 3T3-L1 preadipocytes. Proc. Natl. Acad. Sci. USA.

[44] Yin J, Gao Z, Liu D, Liu Z, Ye J (2008). Berberine improves glucose metabolism through induction of glycolysis. Am. J. Physiol. Endocrinol. Metab..

