# Supplementary material for: Pentamethylquercetin induces adipose browning and exerts beneficial effects in 3T3-L1 adipocytes and high-fat diet-fed mice
Source: Sci Rep. 2017 Apr 25;7:1123. doi: 10.1038/s41598-017-01206-4 (PMC5430711; doi:10.1038/s41598-017-01206-4)
Supplement: Supplementary file 1 — Effects of PMQ on 3T3-L1 adipocytes and mouse gene-specific primers for PCR [file 41598_2017_1206_MOESM1_ESM.pdf]

**Pentamethylquercetin induces adipose browning and exerts beneficial effects in 3T3-L1 adipocytes and high-fat diet-fed mice**

Yi Han, Jian-Zhao Wu, Ji-zhong Shen, Lei Chen, Ting He, Man-wen Jin, Hui Liu

Supplementary materials

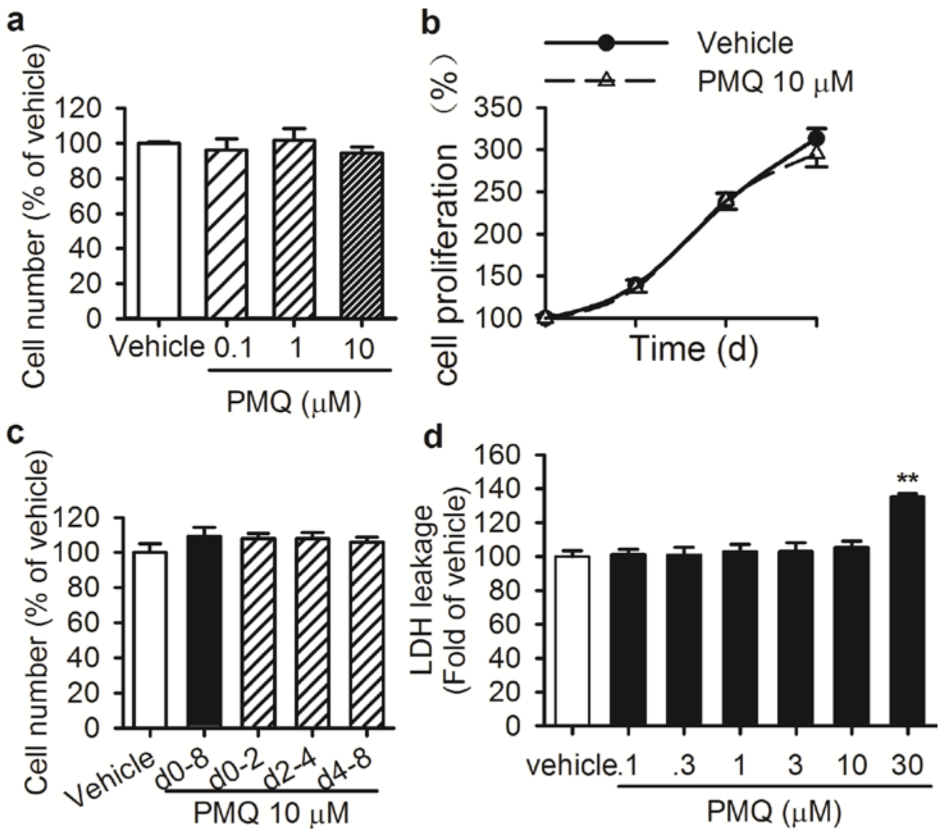


**Figure S1. Effects of PMQ on cell viability in 3T3-L1 adipocytes.** (**a**) 3T3-L1 preadipocytes were treated with PMQ at concentrations of 0.1, 1 and 10 μM for 72 h. (**b**) 3T3-L1 preadipocytes were incubated with 10 μM PMQ for 24, 48, 72 h. (**c**) During the indicated periods (Days 0-8, 0-2, 2-4, 4-8) of 3T3-L1 adipocyte differentiation, the cells were exposed to 10 μM PMQ. Cell proliferation was evaluated by MTT assay on day 8. (**d)** 3T3-L1 preadipocytes were treated with PMQ at concentrations of 0.1, 0.3, 1, 3, 10 and 30 μM for 72 h. Cell toxicity was evaluated by LDH release assay. The data are expressed as the mean ± SEM. *n*=3 experiments, ***p*<0.01 vs vehicle.

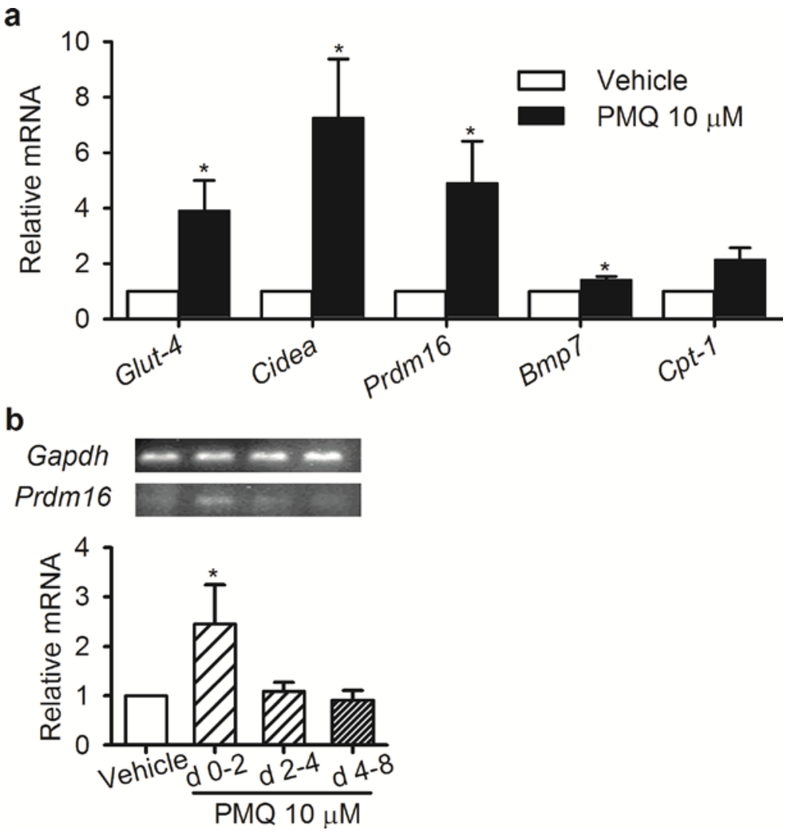


**Figure S2. Effects of PMQ on related genes in 3T3-L1 adipocytes.** (**a**) qPCR analysis of the mRNA levels of the following genes in 3T3-L1 adipocytes: *Glut-4*, *Cidea*, *Prdm16*, *Bmp7* and *Cpt-1*. (**b**) The relative mRNA levels of *Prdm16*. The data are expressed as the mean ± SEM. *n*=3 experiments, **p*<0.05 vs vehicle.

| | Time for cells treated with PMQ | Time for cells treated with DMSO |
|---|---|---|
| Group Vehicle | – | Days 0-8 (a total of 8 days) |
| Group d0-8 | Days 0-8 (a total of 8 days) | – |
| Group d0-2 | Days 0-2 (a total of 2 days) | Days 2-8 (a total of 6 days) |
| Group d2-4 | Days 2-4 (a total of 2 days) | Days 0-2 and Days 4-8 (a total of 6 days) |
| Group d4-8 | Days 4-8 (a total of 4 days) | Days 0-4 (a total of 4 days) |

**Table S1 Time protocol for 3T3-L1 adipocytes in stage-specific experiments**

| | Reverse | Forwad |
|---|---|---|
| *Gapdh* | TGATGTTAGTGGGGTCTCGCTC | GACAAAATGGTGAAGGTCGGTG |
| *Ucp1* | TCGCACAGCTTGGTACGCTT | TACACGGGGACCTACAATGCT |
| *C/ebpα* | CAGTTCACGGCTCAGCTGTT | AAAGCCAAGAAGTCGGTGGA |
| *Pgc-1α* | TTCGCAGGCTCATTGTTGTACTGGT | GGAGCTGGATGGCTTGGGACAT |
| *Pparγ* | CAATCGGATGGTTCTTCGGA | ACTGCCTATGAGCACTTCAC |
| *Prdm16* | AGGAACACGCTACACGGATG | ACTTTGGATGGGAGAGATGCTG |

**Table S2 Mouse gene-specific primers for RT-PCR**

| | Forward | Reverse |
|---|---|---|
| *β-actin* | CTGAGAGGGAAATCGTGCGT | CCACAGGATTCCATACCCAAGA |
| *Ucp1* | ACGGGGACCTACAATGCTTAC | CAGCTTGGTACGCTTGGGTAC |
| *C/ebpα* | GCATCTGCGAGCACGAGA | CCGGGTAGTCAAAGTCACCG |
| *Pgc-1α* | TGTTCCCGATCACCATATTCC | AGCTGTCGTACCTGGGCCTAC |
| *Cidea* | CGTGGTGGACACAGAGGAGTT | CATTGAGACAGCCGAGGAAGT |
| *Bmp7* | TGGACCCCAGAACAAGCAA | CCCTCACAGTAGTAGGCAGCAT |
| *Glut-4* | GCCCCATTCCCTGGTTCA | GGACCCATAGCATCCGCAA |
| *Cpt-1* | TATCGCCACCTGCTGAACC | TTGAAGGTGACGAAGGTGGT |

**Table S3 Mouse gene-specific primers for Q-PCR**
